# ﻿*Reniscymnus* gen. nov., a new genus of Scymnini (Coleoptera, Coccinellidae) from the Oriental region

**DOI:** 10.3897/zookeys.1226.130352

**Published:** 2025-02-06

**Authors:** Feng Peng, Mingjie Tang, Xingmin Wang, Xiaosheng Chen

**Affiliations:** 1 Department of Forest Protection, College of Forestry and Landscape Architecture, South China Agricultural University, Guangzhou 510640, China; 2 Engineering Research Center of Biological Control, Ministry of Education, Guangzhou 510642, China; 3 Department of Entomology, College of Plant Protection, South China Agricultural University, Guangzhou 510640, China

**Keywords:** Biological control, China, Coccinelloidea, identification key, ladybird beetles, Laos, morphology, new species, taxonomy

## Abstract

Ladybird beetles (Coleoptera, Coccinellidae) are a species-rich, ecologically diverse and economically important group of insects. A new ladybird genus of Scymnini, *Reniscymnus***gen. nov.**, is described from China and Laos, along with two new species, *Reniscymnuscordatus* Peng & Chen, **sp. nov.** and *Reniscymnusexplanatus* Peng & Chen, **sp. nov.** This newly identified genus can be distinguished from the remaining members of the tribe Scymnini by its short antenna consisting of eight antennomeres and an inflated antennal club bearing two extremely long setae; the narrow frons; the slightly tapering apically terminal maxillary palpomere; as well as the recurved and laterally almost complete abdominal postcoxal lines that enclose a distinctly wide area. Detailed morphological descriptions, illustrations, and a key to identify these two species within the new genus are provided. Morphological similarities and relationships of the new genus with other genera of Scymnini are discussed.

## ﻿Introduction

The monophyletic family Coccinellidae, more commonly known as ladybirds, ladybugs or ladybird beetles, is the most diverse group among the superfamily Coccinelloidea, with more than 6900 extant species (Wang and Chen 2022). However, after a series of morphological and molecular studies by numerous ladybird taxonomists (Sasaji 1971; Ślipiński 2007; Seago et al. 2011; Che et al. 2021), the evolutionary relationships of major clades within the family remain unresolved. Most recently, molecular phylogenetic studies recognized three subfamilies: Microweiseinae, Monocoryninae and Coccinellinae (Che et al. 2021), with Microweiseinae including about 150 species (Szawaryn et al. 2020), Monocoryninae comprising only 12 species (Szawaryn et al. 2023), and a broadly defined Coccinellinae comprising over 95% of species of ladybirds; although the latter subfamily represents most of ladybird diversity, its internal classification is still poorly understood.

The tribe Scymnini is one of the earliest established taxa within Coccinellidae (Mulsant 1846) and has a complex taxonomic history. Traditionally, the tribe was classified within the former subfamily Scymninae (Mulsant 1850; Sasaji 1971; Gordon 1985; Kovář 1996) or the subfamily Coccinellinae (Korschefsky 1931; Mader 1955; Ślipiński 2007; Seago et al. 2011). Based on comparative morphological studies of adults and larvae, Sasaji (1971) established a comprehensive modern ladybird classification that recognized six subfamilies of Coccinellidae, including the subfamily Scymninae, which consisted of five tribes (Scymnini, Stethorini, Hyperaspini, Aspidimerini and Ortaliini). Since then, almost all modern ladybird taxonomists have regarded Scymnini as a separate subfamily. Recently, Ślipiński (2007) abandoned the tribe Scymnini by allocating all its genera into the tribe Coccidulini among the subfamily Coccinellinae. Subsequently, Seago et al. (2011) also concurred with this placement and synonymized Scymnini with Coccidulini based on molecular data. However, with the addition of more molecular evidence, this placement was not supported and the group was once again treated as an independent tribe (Robertson et al. 2015; Che et al. 2021).

Currently, Scymnini, as a cosmopolitan tribe of ladybirds, comprises 14 genera (Kovář 2007; Wang and Chen 2022; Peng et al. 2023) (namely *Scymnus* Kugelann, 1794; *Nephus* Mulsant, 1846; *Clitostethus* Weise, 1885; *Nephaspis* Casey, 1899; *Scymnobius* Casey, 1899; *Propiptus* Weise, 1901; *Scymniscus* Dorzhansky, 1928; *Acoccidula* Barovskij, 1931; *Axinoscymnus* Kamiya, 1963; *Keiscymnus* Sasaji, 1971; *Leptoscymnus* Iablokoff-Khnzorian, 1978; *Apseudoscymnus* Hoàng, 1982; *Sasajiscymnus* Vandenberg, 2004 and *Slipinskiscymnus* Poorani, Booth & Chen, 2023). Although Kitano (2012) downgraded the genus *Keiscymnus* to a subgenus of *Scymnus*, Peng et al. (2023) did not agree with his arrangement. The tribe accommodates more than 1100 species with high diversity in the northern hemisphere, including high generic diversity in the Oriental region (9 out of 14 genera, including an introduced genus). Historically, Scymnini is a heterogeneous group of ladybirds as it contains numerous genera with superficial morphological similarity based mainly on pubescent body forms, relatively tiny bodies, finely faceted eyes and short antennae. Among them, the largest and cosmopolitan genus, *Scymnus*, is considerably diverse and speciose with more than 800 recognized species (Chen et al. 2015a), as well as small endemic genera with regional distribution (Peng et al. 2022). In addition, there are four monotypic genera (*Acoccidula*, *Apseudoscymnus*, *Leptoscymnus*, and *Propiptus*) that have not been recorded since their original description. The taxonomic status of these genera remains questionable. Moreover, no synapomorphy has been identified to support the monophyly of Scymnini. The group seems to represent a taxonomic dumping ground among the tribes of Coccinellidae, similar to Coccidulini, which is predominantly distributed in Southern Hemisphere (Szawaryn et al. 2021). Furthermore, recent molecular-based analyses showed that the members of the tribe do not form a monophyletic group (Magro et al. 2010; Seago et al. 2011; Robertson et al. 2015; Che et al. 2021). Hence, the taxonomic status, morphological revision and phylogenetic studies of the tribe Scymnini need to be further studied.

In addition, in the last decade, the taxonomy of several genera of Scymnini from the Oriental region has been greatly improved, including *Scymnus* [e.g., Chen et al. (2012, 2013, 2014, 2015a, 2015b, 2015c, 2016)]; *Sasajiscymnus* (Tong et al. 2022); *Axinoscymnus* (Peng et al. 2022); *Slipinskiscymnus* (Peng et al. 2023) and in the tribe generally (Poorani 2015, Poorani and Lalitha 2018, Poorani and Booth 2021), which has contributed to a comprehensive understanding of species diversity and distribution patterns of Scymnini. Species of Scymnini exhibit a broad feeding habit on whiteflies, mealybugs, aphids and scale insects belonging to the most economically important groups; thus, they are potential biological control agents (Wang and Chen 2022).

The present study was inspired by the discovery of a remarkable new genus of the tribe Scymnini, distributed in the Oriental region. This genus is described here as *Reniscymnus* gen. nov., along with the species *R.cordatus* Peng & Chen, sp. nov. and *R.explanatus* Peng & Chen, sp. nov. A key to these two species of the new genus is given.

## ﻿Material and methods

The specimens examined were collected from China and Laos, and were deposited in the collection of the Department of Entomology, South China Agricultural University (**SCAU**), Guangzhou, China. The morphological terminology used in this paper follows Ślipiński (2007). Measurements were carried out using a micrometer attached to a stereomicroscope (SteREO Discovery V20) and are defined as follows: total length (**TL**), from apical margin of clypeus to apex of elytra; total width (**TW**), equal to elytral width (**EW**), across both elytra at widest part; total height (**TH**), at the highest part of the body in lateral view; pronotal length (**PL**), from the middle of anterior margin to the base of pronotum; pronotal width (**PW**), across widest part; elytral length (**EL**), along suture from base to apex including scutellar shield; and head width (**HW**), at the widest part including the eyes.

External morphology was examined using a stereomicroscope (Stemi 508, ZEISS). Male and female genitalia were dissected, cleared in a 10% NaOH solution, and placed on slides for further study. The illustrations of adults were captured with a digital camera (EOS 5D Mark IV, Canon) mounted on a focus stacking rail (WeMacro Rail) and controlled by the software Helicon Remote v. 3.9.12. Genitalia were photographed using a digital camera (Axiocam 506 color) attached to a microscope (Imager M2, ZEISS) and controlled by the software ZEN v. 2.3. After examination and photography, the genitalia were glued on a card and pinned beneath the specimen. Finally, the software Helicon Focus v. 8.1.1 and Adobe Photoshop 2020 were used to render and clean up images, respectively.

## ﻿Results

**Order Coleoptera Linnaeus, 1758**

**Family Coccinellidae Latreille, 1807**

**Subfamily Coccinellinae Latraille, 1807**

**Tribe Scymnini Mulsant, 1846**

### ﻿ Reniscymnus

Taxon classificationAnimaliaColeopteraCoccinellidae

Peng & Chen gen. nov.

B0C39653-82A5-555C-BAE5-E016485CC482

https://zoobank.org/96D790DC-E1BB-46E2-B948-D198BDC84C8D

#### Type species.

*Reniscymnuscordatus* Peng & Chen, sp. nov.

#### Etymology.

The generic name is derived from the generic name *Scymnus* and the memory of the late Prof. Ren Shunxiang from South China Agricultural University, a well-known Chinese entomologist who devoted most of his life to the study of Coccinellidae and biological control. Gender masculine.

#### Diagnosis.

**Figure 1.**
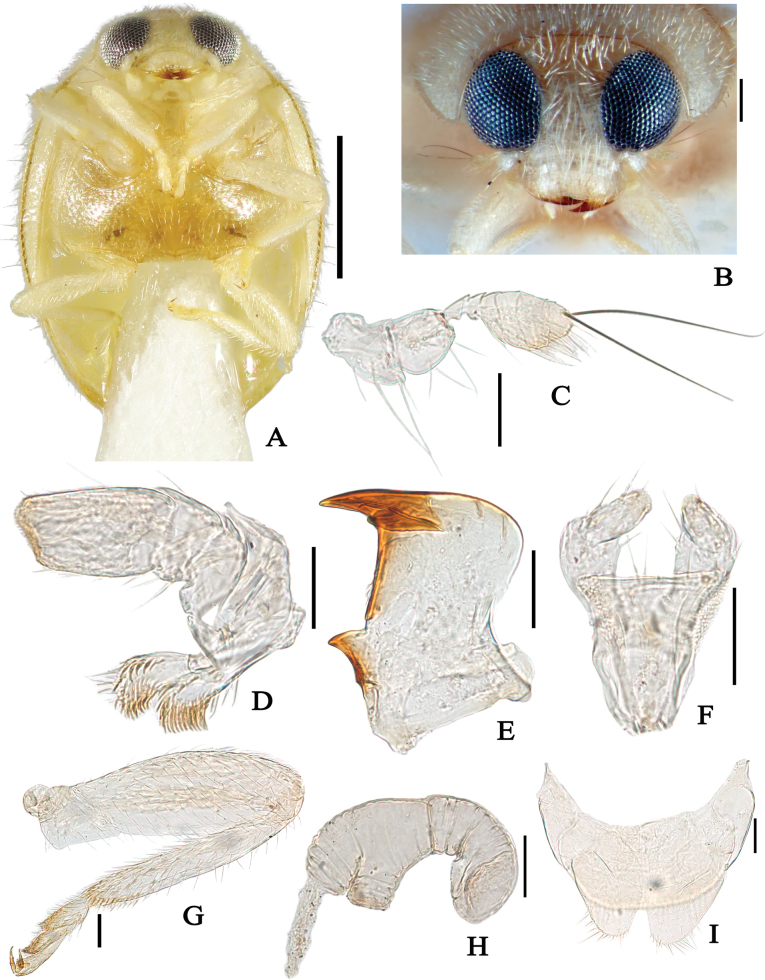
Main characters of the genus *Reniscymnus* gen. nov. **A**–**I***Reniscymnuscordatus* Peng & Chen, sp. nov. **A** ventral view **B** head **C** antenna **D** maxilla **E** mandible **F** labium **G** hind leg **H** spermatheca **I** coxites. Scale bars: 0.5 mm (**A**); 0.1 mm (**B**); 0.05 mm (**C–I**).

**Figure 2.**
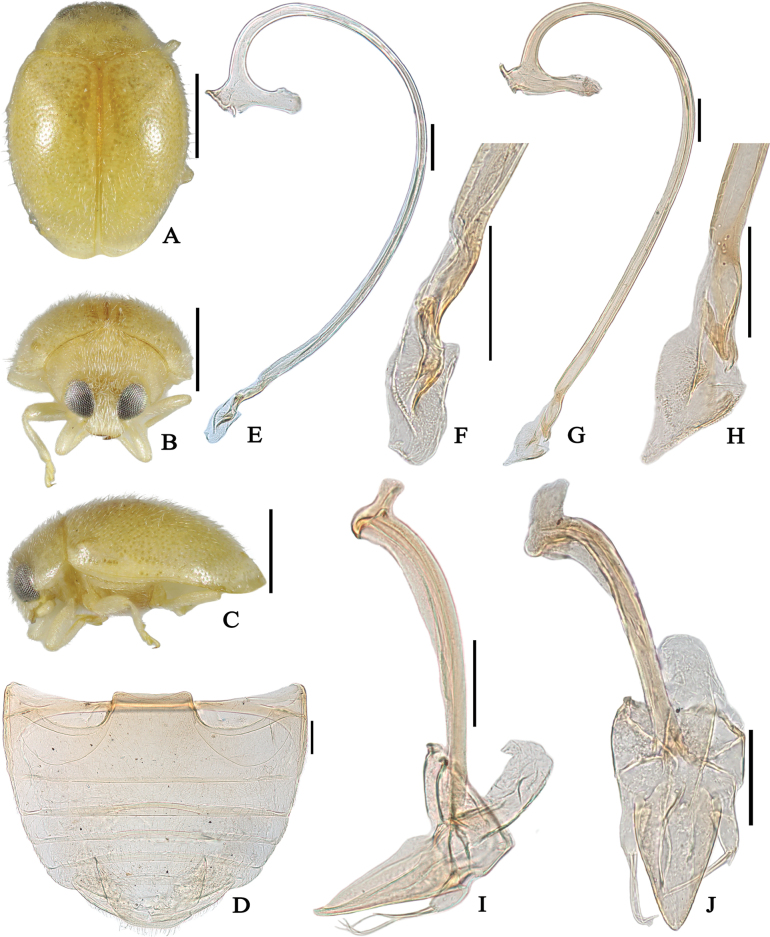
*Reniscymnuscordatus* Peng & Chen, sp. nov. (**A–F, I, J** holotype **G, H** paratype) **A** dorsal view **B** frontal view **C** lateral view **D** abdomen **E, G** penis **F, H** apex of penis **I** tegmen, lateral view **J** tegmen, inner view. Scale bars: 0.5 mm (**A–C**); 0.1 mm (**D–J**).

**Figure 3.**
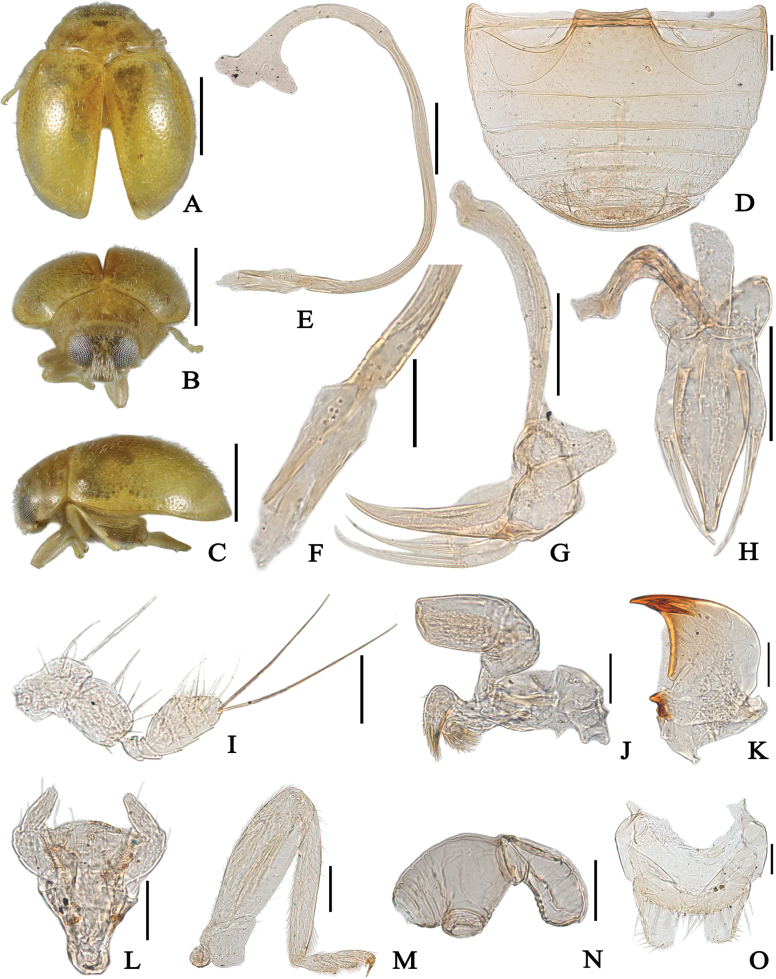
*Reniscymnusexplanatus* Peng & Chen, sp. nov. (**A–H** holotype **I–O** paratype) **A** dorsal view **B** frontal view **C** lateral view **D** abdomen **E** penis **F** apex of penis **G** tegmen, lateral view **H** tegmen, inner view **I** antenna **J** maxilla **K** mandible **L** labium **M** hind leg **N** spermatheca **O** coxites. Scale bars: 0.5 mm (**A–C**); 0.1 mm (**D–H**); 0.05 mm (**I–O**).

*Reniscymnus* gen. nov. presents some characters found in other genera of Scymnini (especially *Sasajiscymnus*, *Slipinskiscymnus* and *Axinoscymnus*). But the new genus can be easily distinguished from the latter by the following characters: frons narrow, less than the width of an eye (Fig. 1A) (vs. wide, more than the width of an eye in *Sasajiscymnus* and *Slipinskiscymnus*); antenna composed of 8 antennomeres with a distinctly swollen scape and pedicel, antennomeres 5–8 forming a fusiform and inflated club and the terminal antennomere with two extremely long setae (Figs 1C, 3I) (vs. 9 antennomeres in *Sasajiscymnus*; 10 antennomeres in *Slipinskiscymnus*, and 11 antennomeres in *Axinoscymnus*); terminal maxillary palpomere slightly narrows apically (Figs 1D, 3J) (vs. expanded apically in *Sasajiscymnus*; *Slipinskiscymnus* and *Axinoscymnus*); prosternal process with carinae slightly convergent anteriorly (Fig. 1A) (vs. complete carinae parallel, joined anteriorly forming square in *Sasajiscymnus*; narrow carinae in *Axinoscymnus*); abdominal postcoxal lines incomplete, strongly recurved toward the base of ventrite 1 but not reaching it, the area enclosed by abdominal postcoxal lines distinctly wide (Figs 2D, 3D) (vs. incomplete, generally parallel to the posterior margin of ventrite 1 in *Sasajiscymnus*; complete in *Slipinskiscymnus* and *Axinoscymnus*); penis guide subconical with a pointed apex (Fig. 2I–J); parameres rather short with few long setae (Fig. 2I–J).

#### Description.

Body elongate oval, color lighter and yellowish, dorsum moderately convex, covered with uniform short setae (Fig. 2A–C).

Head transverse; frons relatively narrow, less than the width of an eye (Fig. 1A). Eyes large, rounded without eye canthus, finely faceted with short interfacetal setae; inner ocular margin arcuate (Fig. 1B). Clypeus short, and expanded laterally, not covering antennal insertions, anterior margin slightly convex (Fig. 1B). Labrum exposed. Antennae short with 8 antennomeres (Figs 1C, 3I); scape stout, widened and curved apically with several long setae; pedicel longest, swollen and barrel-shaped, as wide as scape; scape and pedicel combined nearly as long as antennomeres 3–8; antennomere 3 distinctly narrowed at base, recurved and turgidly expanded apically; antennomere 4 recurved, expanded apically; antennomeres 5–8 forming fusiform and inflated club with many short setae, and terminal antennomere trochiform with two extremely long setae (Figs 1C, 3I). Mandibles bifid apically, with well-developed molar tooth (Figs 1E, 3K). Maxillary palp 4-segmented; palpomere 1 short, ring-like; palpomere 2 elongate, widened apically; palpomere 3 subtriagular and compact at inner margin; terminal palpomere slightly narrowing apically, apical margin slightly obliquely truncate (Figs 1D, 3J). Maxilla with cardo round and laterally not expanded. Mentum narrowed basally, parallel-sided in basal third, widened apically, anterior margin of mentum weakly arcuate (Figs 1F, 3L). Labial palp 3-segmented, terminal palpomere nearly as long as penultimate one (Figs 1F, 3L).

Pronotum transverse with anterior corners roundly produced. Scutellar shield triangular and distinctly tiny (Fig. 2A). Elytra with humeral calli distinct, with apex sharply rounded. Prosternum with narrow procoxae anteriorly, prosternal process wide and subquadrate, carinae widely separated, slightly convergent anteriorly, reaching anterior prosternum margin (Fig. 1A). Mesoventrite transverse with a truncate anterior margin; Mesocoxal process wide, slightly wider than width of corresponding mesocoxal diameter. Meso-metaventral junction visible, forming straight line. Metaventrite weakly convex, metaventral postcoxal lines widely separate medially, roundly recurved and complete laterally (Fig. 1A). Discrimen visible. Elytral epipleuron incomplete, reaching posterior margin of abdominal ventrite 1, without foveae (Fig. 1A).

Legs short, extending a little beyond lateral margins of elytra; trochanters wide, deviously and angulately produced externally; femora relatively slender; tibiae rather slender, about 1/2 as wide as femur, without apical spurs; tarsi with 3 tarsomeres, tarsomeres 1 and 2 lobed, tarsal claws with sharp basal tooth (Figs 1G, 3M).

Abdominal processes transverse, distinctly wide. Ventrite 1 distinctly longer than ventrite 2, in middle almost 2 times length of ventrite 2 (Figs 2D, 3D). Abdomen with six ventrites in both sexes. Abdominal postcoxal lines incomplete, strongly recurved toward base of ventrite 1 but not reaching it, posteriorly extending more than half of length of ventrite 1 (Figs 2D, 3D), area enclosed by abdominal postcoxal lines distinctly wide.

Male genitalia. Relatively long, penis slender (Fig. 2E); apex of penis modified with membranous appendages (Fig. 2F); tegminal strut stout with a finger-like protrusion at apex. Penis guide stout, subconical with a pointed apex in inner view, parameres rather short with few long setae (Fig. 2I–J).

Female genitalia. Coxites shortened without styli (Figs 1I, 3O); spermatheca short and recurved with shortened cornu and swollen ramus and nodulus (Figs 1H, 3N).

#### Distribution.

China (Hainan, Guangdong, Guangxi, Yunnan); Laos.

#### Remarks.

The species described in this study are distributed in the Oriental region. Interestingly, both new species of this genus exhibit a lighter body coloration similar to that of *Axinoscymnuscardilobus* Ren & Pang, 1992, which is in contrast to the darker or black body coloration among members of the tribe Scymnini.

### ﻿Key to the species of the genus *Reniscymnus* gen. nov.

**Table:** 

1	Penis longer than abdomen, penis capsule with long and well-developed inner arm, apex of penis swollen; penis guide straight in lateral view, penis guide widest at base in inner view	***Reniscymnuscordatus* Peng & Chen, sp. nov.**
–	Penis shorter than abdomen, penis capsule with inner arm slightly reduced, apex of penis flattened; penis guide slightly recurved at apex in lateral view, penis guide constricted at base, widest in basal 1/3 in inner view	***Reniscymnusexplanatus* Peng & Chen, sp. nov.**

### ﻿ Reniscymnus cordatus

Taxon classificationAnimaliaColeopteraCoccinellidae

Peng & Chen sp. nov.

743646CE-001E-5F37-8DBB-CAE2B8CE5FBD

https://zoobank.org/97976073-3815-4C0A-8E2F-1B30E7CE7DA6

Fig. 2

#### Diagnosis.

This species resembles *R.explanatus* sp. nov. in external appearance but differs from the latter in the well-developed and elongate inner arm of penis capsule, and the subtriangular tegmen with penis guide in lateral view.

#### Description.

TL: 1.38–1.63 mm, TW: 1.08–1.25 mm, TH: 0.70–0.78 mm, TL/TW: 1.26–1.41, EL/EW: 0.99–1.09, PL/PW: 0.49–0.52, HW/PW: 0.64–0.67, PW/EW: 0.66–0.69.

Body elongate oval, dorsum with white pubescence. Area of frons near clypeus with suberect and downward setae, distinctly longer and denser than those on top (Fig. 2B). Body uniformly pale yellow, except tip of mandibles dark brown (Fig. 2A–C). Eyes finely faceted, large, interocular distance about 0.3 times head width. Head and pronotal punctures fine and dense. Surface of elytra with coarse and shallowly impressed punctures.

Abdominal postcoxal lines incomplete and roundly recurved, reaching 3/4 of length of ventrite 1, area enclosed by the lines sparely punctate, widely smooth along lines; ventrite 1 with fine and sparse punctures in middle, irregularly distributed (Fig. 2D).

Male genitalia. Penis moderately stout and rather long, longer than abdomen; penis capsule with long inner arm and short outer arm (Fig. 2E, G), apex of penis swollen with cordate membranous appendages (Fig. 2F, H); tegminal strut stout with a finger-like protrusion at apex (Fig. 2I–J); phallobase subtrapezoid in lateral view (Fig. 2I); tegmen with penis guide triangular, widest at base, apex pointed in lateral view, 3.5 times as long as parameres (Fig. 2I); in inner view, penis guide triangular, gradually converging apically; parameres short with few long setae at apex (Fig. 2J).

#### Type material.

***Holotype***: Laos • 1♂; Phou Kbouay NBCA; alt. 500–750 m; 2–4 Jun. 2007; X.M. Wang et al. leg.; No.SCAU(E)17549; SCAU. ***Paratypes***: 1♂; same data as for holotype • 1♂1♀; Vieng Xai, Xam Nua; alt. 1000 m; 11–12 Jun. 2007; X.M. Wang et al. leg. • 1♀; Heu Gnommolat; alt. 380 m; 25 May 2007; X.M. Wang et al. leg. • 2♀; Tad Fane, Pakxong; 11 Jun. 2006; C. Telakang leg. • 1♀; LiPHi, VoeunKHom; 9 Jun. 2006; C. Telakang leg. • 1♀; Khammouane Lakxao; 16 Dec. 2005; C. Telakang leg. China – **Hainan Prov.** • 1♂1♀; Xin’an, Diaoluoshan Mountains; 18 Sep. 1995; Z.Q. Peng leg. • 1♂; Jianfengling; 1 Apr. 1996; Z.Q. Peng leg. • 2♂4♀; Tianchi, Jianfengling; Sep. 1995; Z.Q. Peng leg. • 2♂8♀; Wuzhishan Mountains; 3 May 1996; Z.Q. Peng leg. • 1♂; Shamaoling, Danzhou City; Aug. 1995; Z.Q. Peng leg. • 1♂; Shijing, Diaoluoshan Mountains; Sep. 1995; Z.Q. Peng leg. • 1♀; Wufenchang, Limushan Mountains; Sep. 1995; Z.Q. Peng leg. • 1♀; Limushan Mountains; 21 Apr. 1996; Z.Q. Peng leg. • 1♂; Yinggezui, Yinggeling; 16 Apr. 2019; X.M. Wang leg. • 1♀; Wuzhishan Forest Park; 18°54'26"N, 109°40'47"E; alt. 670 m; 16 Apr. 2019; X.M. Wang leg. • 1♀; Bawangling; 16 Oct. 1997; Z.Q. Peng leg. • 1♀; Limushan Mountains; 22 Jul. 2006; Z.Q. Peng leg. – **Guangxi Prov.** • 1♂1♀; Fulongshan Mountains, Shiwandashan Mountains; 7 Nov. 2004; C.W. Zhang leg. • 2♀; Daqingshan Mountains, Pingxiang; 2 Aug. 2005; X.M. Wang leg. • 1♀; Banshanyao, Mao’ershan Mountains; 16 Oct. 2004; X.M. Wang leg. – **Guangdong Prov.** • 1♂3♀; Nankunshan Mountains, Huizhou; 21 Dec. 2004; Collector unknown. • 1♂; Wuzhishan Scenic Spot, Liangkou Town, Conghua District, Guangzhou City; 23°43'14"N, 113°48'52"E; alt. 433 m; 16 Mar. 2024; Z.D. Huang et al. leg. – **Yunnan Prov.** • 2♀; Tongbiguan, Nabang, Yingjiang; alt. 1000 m; 22–23 May 2008; X.M. Wang et al. leg. • 1♀; Xiaoweishan Mountains, Hekou; 23 Apr. 2008; X.M. Wang et al. leg.

#### Distribution.

China (Hainan, Guangdong, Guangxi, Yunnan); Laos.

#### Etymology.

The species epithet is derived from the Latin adjective “*cordatus*”, referring to the nearly cardioid apex of the penis.

#### Remarks.

The apex of the penis of this species is spatially swollen; consequently, different shapes were observed when the apex was rotated or viewed from different angles (Fig. 2E–H).

### ﻿ Reniscymnus explanatus

Taxon classificationAnimaliaColeopteraCoccinellidae

Peng & Chen sp. nov.

8EE0B488-CC19-5C47-AEFE-070527DD1C64

https://zoobank.org/0BF770A6-FD04-4675-BDCC-271E0124A9FD

Fig. 3

#### Diagnosis.

This species is similar to *Reniscymnuscordatus* sp. nov. in colour pattern, but can be distinguished from the latter by the relatively shortened inner arm of penis, flattened apex of penis, and the unique tegmen with penis guide recurved inwardly at apex in lateral view and constricted at base in inner view.

#### Description.

TL: 1.51–1.65 mm, TW: 1.13–1.21 mm, TH: 0.70–0.83 mm, TL/TW: 1.33–1.36, EL/EW: 1.05–1.07, PL/PW: 0.50–0.51, HW/PW: 0.66–0.68, PW/EW: 0.66–0.69.

Body rounded oval, dorsum with white pubescence. Area of frons near clypeus with suberect and downward setae, distinctly longer and denser than those on top (Fig. 3B). Body yellow, except tip of mandibles dark brown, metaventrite deep yellow in middle and elytral epipleurae brown (Fig. 3A–C). Eyes finely faceted, large, interocular distance about 0.33–0.34 times head width. Head and pronotal punctures fine and dense. Surface of elytra with coarse, shallowly impressed punctures.

Abdominal postcoxal lines incomplete and roundly recurved, reaching 5/6 length of ventrite 1, area enclosed by the lines sparsely punctate, widely smooth along the lines; ventrite 1 with coarse and sparse punctures in middle, irregularly distributed (Fig. 3D).

Male genitalia. Penis stout and relatively short, shorter than abdomen; penis capsule with short inner arm, slightly longer than outer arm (Fig. 3E); apex of penis wide, flattened with few membranous appendages (Fig. 3F); tegminal strut stout with finger-like protrusion at apex (Fig. 3G); phallobase rounded in lateral view; tegmen with penis guide widest at base, gradually converging apically, slightly recurved at apex in lateral view, 3 times as long as parameres (Fig. 3G); in inner view, penis guide slightly constricted at base, widest in basal 1/3, then gradually tapering apically, apex blunt (Fig. 3H); parameres short with several long setae at apex (Fig. 3H).

#### Type material.

***Holotype***: Laos • ♂; Pakxong; alt. 1280 m, 24 May 2007; X.M. Wang et al. leg. No. SCAU(E)17561; SCAU. ***Paratypes***: • 1♀; same data as for holotype • 1♂; Heu Gnommolat; alt. 380 m; 25 May 2007; X.M. Wang et al. leg. • 1♀; Phou Khao Kbouay NBCA; alt. 500–750 m; 2–4 Jun. 2007; X.M. Wang et al. leg. • 5♂9♀; Khammouane Tnangend; 21 Dec. 2005; C. Telakang leg. • 1♂; Khammouane Lakxao; 16 Dec. 2005; C. Telakang leg. • 1♀; Khammouane Thakhex; 25 Dec. 2005; C. Telakang leg.

#### Distribution.

Laos.

#### Etymology.

The species epithet is derived from the Latin adjective “*explanatus*”, referring to the flattened apex of the penis.

## ﻿Discussion

The two species have similar external appearance and male genitalia. A generally consistent set of characters support their placement within the tribe Scymnini: (1) dorsum densely pubescent; (2) antennae distinctly shorter than half width of head; (3) eye finely faceted; (4) clypeus not covering antennal insertions, only slightly expanded laterally; and (5) tegmen and penis similar to other members of Scymnini.

The new genus shows considerable similarity to *Axinoscymnus*, as some members of the genus are characterized by the yellow in colour pattern and structure of extremely large eyes and narrow frons. However, in *Axinoscymnus*, the antenna is composed of eleven antennomeres, terminal maxillary palpomeres are slightly broadened, the metaventrite is strongly convex, abdominal postcoxal lines are complete and male genitalia are short with the penis capsule distinctly reduced. In *Reniscymnus* gen. nov., the antenna is composed of eight antennomeres, metaventrite is slightly convex, abdominal postcoxal lines are incomplete, and male genitalia is elongate and robust with a well-developed penis capsule.

Among the Oriental genera of Scymnini, *Reniscymnus* gen. nov., *Sasajiscymnus* and *Slipinskiscymnus* share trimerous tarsi and antenna with a compact and broad club. In addition, the new genus also shares its closest affinity with *Sasajiscymnus* due to both genera exhibiting similarities such as two long setae positioned in their terminal antennomere and comparable male genitalia with the robust penis guide and thin parameres. The new genus particularly resembles species within the *Sasajiscymnushareja* species group (Tong et al. 2022), which possesses a penis guide at the base with a prominent keel in lateral view in males, and a pair of elongate coxites with well-developed styli and spermatheca with relatively elongate cornu in female. On the other hand, in *Reniscymnus* gen. nov., the penis guide has a wide base without a prominent keel in lateral view in the male, while the coxites are shortened and without styli and spermatheca with distinctly shortened cornu. Moreover, in *Reniscymnus*, the antenna comprises eight antennomeres. However, *Sasajiscymnus* has nine antennomeres, while *Slipinskiscymnus* possesses ten antennomeres.

Additionally, in *Reniscymnus*, the abdominal postcoxal lines are incomplete, strongly recurved toward the base of ventrite 1 but not reaching it and the area enclosed by the abdominal postcoxal lines is distinctly wide. Conversely, in *Sasajiscymnus*, the abdominal postcoxal lines are incomplete and generally parallel to the posterior margin of ventrite 1. In very rare cases, these lines may slightly recurve apically or strongly curve and extend to the base as in *Sasajiscymnusnepalicus* (Miyatake, 1985) and *S.pronotus* (Pang & Huang, 1986); however, in *Slipinskiscymnus*, the abdominal postcoxal lines are recurved and complete. Nonetheless, *Slipinskiscymnus* also demonstrates the prosternal process with anteriorly convergent carinae similar to that in *Reniscymnus* gen. nov.; furthermore, *Slipinskiscymnus* has the male genitalia with the asymmetrical apex of the penis guide in inner view (symmetrical in *Reniscymnus*) and also having the distinct lateral expansion (while the maxillary cardo of *Reniscymnus* has no expansion). Besides, in *Sasajiscymnus*, the prosternal process has a complete carinae that runs parallel before joining anteriorly and forming sides of a square shape.

Moreover, in both *Sasajiscymnus* and *Slipinskiscymnus*, the terminal maxillary palpomere shows a slight broadening compared to its slightly narrow counterpart found in *Reniscymnus*. Finally, *Reniscymnus* gen. nov. is distinguished from both *Slipinskiscymnus* and *Sasajiscymnus* based on its laterally expanded clypeus and narrower frons, which measures less than eye width.

In *Reniscymnus* gen. nov., the apically narrowed terminal maxillary palpomere is similar to that in *Keiscymnustosaensis* Sasaji (type species of the genus). However, the latter is distinct from the new genus in its distinct shortened terminal labial palpomeres, 10-segmented antenna without long setae, complete abdominal postcoxal lines, and male genitalia with well-developed lobed parameres.

## Supplementary Material

XML Treatment for Reniscymnus

XML Treatment for Reniscymnus cordatus

XML Treatment for Reniscymnus explanatus

## ﻿Additional information

### Author ORCIDs

Feng Peng https://orcid.org/0000-0002-7797-5709

Xingmin Wang https://orcid.org/0000-0001-9327-9159

Xiaosheng Chen https://orcid.org/0000-0001-8253-4943

### Data availability

All of the data that support the findings of this study are available in the main text.
